# AI-Driven Food Fraud Detection Systems: A Critical Systematic Review of the Detection–Prevention Gap

**DOI:** 10.3390/foods15183185

**Published:** 2026-09-09

**Authors:** Orlando Meneses Quelal, David Pilamunga Hurtado, Marco Burbano Pulles

**Affiliations:** Carrera de Alimentos, Universidad Politécnica Estatal del Carchi, Tulcán 040101, Ecuador; luis_david19lp@hotmail.com (D.P.H.); marco.burbano@upec.edu.ec (M.B.P.)

**Keywords:** food fraud, adulteration, artificial intelligence, machine learning, deep learning, NIR spectroscopy, electronic nose

## Abstract

The economic impact of food fraud is difficult to quantify precisely, because fraud is structurally designed to evade detection; available estimates are indirect projections rather than direct forensic accounting and are commonly cited in the range of USD 10–15 billion annually. The integration of artificial intelligence (AI) with analytical instrumentation has generated a rapidly expanding body of research aimed at detecting adulteration, mislabeling, and substitution across food matrices. This systematic review examines the extent to which AI-assisted instrumental technologies contribute to food fraud prevention (as distinct from laboratory detection) and characterizes the structural factors that constrain real-world translation. A systematic search of the peer-reviewed literature published between 2021 and 2026 yielded 83 eligible records (80 primary studies and 3 review articles) after applying predefined inclusion criteria. Data were extracted into a structured seven-sheet workbook covering study characteristics, instrumental technologies, AI architectures, performance metrics, industrial-validation status, implementation evidence, and methodological quality. The corpus shows consistently high reported analytical accuracy under controlled laboratory conditions (median of extractable classification accuracies ≈ 99–100%; ≥95% in 86% of studies with an extractable value). At the same time, 68 of 83 studies (82%) reported no external validation, no study (0/83) achieved inter-laboratory validation, no study documented routine-monitoring application, and only one study reported testing in a genuine industrial environment. The most frequently featured platforms were NIR spectroscopy and electronic-nose arrays (each featuring in 30/83 studies, frequently in data-fusion combinations), followed by gas-chromatography-based systems (16/83) and hyperspectral imaging (13/83). Classical machine learning predominated (57/83 studies coded as classical ML, with a further 11 hybrid ML/DL designs and 12 deep-learning-only designs). A direct statistical comparison found no significant difference in reported accuracy between classical-ML and deep-learning studies (median 100% vs. 98.2%; Mann–Whitney U test, *p* = 0.16). A pre-specified test of the hypothesis that high reported accuracy is itself a marker of overfitting was not supported by the corpus: reported accuracy was not negatively associated with external-validation status (Fisher’s exact *p* = 0.51) or with methodological-quality score (Spearman ρ = 0.15, *p* = 0.23). Methodological quality was predominantly moderate (49/83 scored 3/5; 22 scored 2/5; 11 scored 4/5; one study scored 5/5), and 19/83 (23%) carried a high risk of bias. The review’s central observation—a measurable gap between demonstrated laboratory detection and evidenced real-world prevention—is well supported by the deployment, inter-laboratory, and routine-monitoring data. We deliberately separate this strongly evidenced conclusion from weaker inferences (e.g., the overfitting hypothesis) that the corpus cannot currently establish, and we outline a validation-driven, deployment-oriented research agenda.

## 1. Introduction

### 1.1. The Persistent Problem of Food Fraud

Food fraud—defined as the deliberate and intentional substitution, addition, tampering, or misrepresentation of food, food ingredients, or food packaging for economic gain—constitutes one of the most complex challenges confronting contemporary food safety governance. Unlike food safety failures resulting from unintentional contamination, food fraud is characterized by intentionality, economic motivation, and systematic evasion of existing detection mechanisms. Its manifestations range from geographic origin misrepresentation [1,2,3] and species substitution [4,5,6,7] to adulteration with chemically similar compounds [8,9,10,11], dilution with economically motivated adulterants [12,13,14,15], and addition of unauthorized synthetic compounds [16,17,18].

Global estimates place annual costs in the order of USD 10–15 billion [19], with cascading effects on consumer health, trade, and regulatory credibility. High-value commodities—olive oil, honey, spices, premium teas, dairy, and edible oils—are disproportionately targeted, a pattern mirrored by the matrices that dominate the present corpus (oils and fats, dairy, teas, spices, and honey) [9,10,14,15,20,21,22,23].

The detection challenge is compounded by the increasing chemical sophistication of fraudulent practices. Geographic-origin fraud exploits overlapping chemical profiles across origins (e.g., soybean origin classification [1]); substitution of premium oils with cheaper vegetable oils requires methods that resolve chemically similar lipid profiles (e.g., camellia oil [11,24]); and adulteration of teas with synthetic pigments at concentrations as low as 0.1% [16] demands analytical sensitivity near regulatory margins. These pressures have driven substantial investment in AI-assisted analytical systems.

### 1.2. The Rise of AI-Assisted Detection

The 2021–2026 period shows an accelerating convergence of analytical chemistry, instrumental spectroscopy, and machine learning in food authenticity. Publication volume in this corpus grew from two records in 2021 to 36 in 2025, with 16 already indexed in the first months of 2026. The most frequently featured analytical platform is NIR spectroscopy [2,9,10,15,17,23,25,26,27,28], valued for non-destructive, rapid, and relatively low-cost measurement; it is complemented by electronic-nose arrays [1,3,8,12,29,30,31,32,33,34], hyperspectral imaging, and increasingly by multi-sensor data-fusion architectures [4,11,28,31,34,35,36].

Classical algorithms—Support Vector Machines (SVM), Random Forest (RF), and Artificial Neural Networks (ANN)—dominate the methodological landscape, while deep-learning architectures (CNNs, Transformers, and hybrids) form a smaller but growing share [1,6,11,18,24,30,35,37,38,39,40,41,42,43,44]. Reported performance is consistently high, and this has generated considerable optimism about AI’s potential to transform fraud detection. As shown in Section 3, however, this optimism is concentrated almost entirely in internal performance, with limited evidence on external or operational performance.

### 1.3. The Central Research Question and Its Conceptual Frame

Despite analytical optimism, one question remains systematically under-addressed: to what extent do these systems contribute to the prevention of food fraud in real supply chains? Detection under controlled laboratory conditions—authenticated reference samples, known adulterant concentrations, single origins, and optimized instruments—is methodologically tractable. Prevention additionally requires validation across the variability of real supply chains, integration into enforcement workflows, economic viability for routine deployment, and robustness across instruments, laboratories, seasons, and origins.

This review addresses three questions: (1) What is the actual state of external validation and independent-dataset use in AI-driven food-fraud detection research? (2) What is the evidence for industrial implementation and regulatory integration? (3) What structural factors plausibly explain any gap between laboratory performance and real-world prevention impact? We treat (1) and (2) as primarily empirical questions answerable from the workbook, and (3) as an interpretive question on which we report both supporting and countervailing evidence.

### 1.4. From Analytical Accuracy to Decision-Theoretic Utility

A frequently overlooked dimension is the distinction between analytical accuracy and decision-theoretic utility. Metrics such as accuracy, AUC, or R^2^ quantify predictive performance under controlled conditions, but they do not directly translate into actionable decisions in regulatory or industrial environments, where detection operates under asymmetric error costs: false negatives may allow fraudulent products into the supply chain, whereas false positives may cause unnecessary economic loss and trade disruption. The operational value of a detector depends on the prior probability of fraud in a given context and on the cost structure of errors—parameters that, in this corpus, are not explicitly modeled in any study. This is a genuine conceptual limitation; we are careful, however, not to overstate it as evidence that deployed performance would necessarily collapse, since the corpus provides little direct measurement either way.

### 1.5. Objectives

The primary objective is to assess the contribution of AI-assisted instrumental technologies to food-fraud prevention and to characterize the limitations constraining translation from laboratory proof-of-concept to operational deployment. Secondary objectives are to characterize the distribution of analytical technologies and AI architectures; quantify the frequency and rigor of external validation; map the landscape of industrial and regulatory implementation evidence; statistically compare classical-ML and deep-learning performance; test directly whether high reported accuracy is associated with weaker validation; and evaluate methodological quality using a structured risk-of-bias framework.

## 2. Methods

### 2.1. Protocol and Registration

The review was conducted in accordance with the PRISMA 2020 guidelines for systematic reviews and meta-analyses. To ensure methodological transparency and reproducibility, the review protocol was prospectively deposited in Zenodo prior to study selection and data extraction (DOI: 10.5281/zenodo.20785718). The protocol predefined the research questions, eligibility criteria, search strategy, data extraction framework, methodological quality assessment procedures, and planned statistical analyses. The final database search was conducted on 12 June 2026.

### 2.2. Eligibility Criteria

Studies were eligible if they (1) reported at least one quantitative performance metric (accuracy, precision, recall, F1, AUC, R^2^, RMSE, or equivalent); (2) applied one or more AI/ML methods to a food-authentication, adulteration-detection, or fraud-related classification or regression problem; (3) described the instrumental technology used for data acquisition; (4) were published in peer-reviewed journals between January 2021 and June 2026; and (5) were available in English or provided sufficient tabular/graphical data for extraction. Review articles were retained when they offered meta-level analytical insight relevant to the review question. Studies were excluded if they addressed food-safety problems unrelated to fraud, reported only in vitro work, were conference abstracts lacking methodological detail, or addressed post-market surveillance without an AI modeling component.

### 2.3. Search Strategy and Study Selection

The search spanned Scopus/ScienceDirect, PubMed, IEEE Xplore, and MDPI (publisher platform searched supplementarily) databases using combinations of terms, including ‘food fraud detection’, ‘food adulteration’, ‘food authentication’, ‘machine learning food’, ‘deep learning food fraud’, ‘NIR spectroscopy adulteration’, ‘electronic nose food’, ‘hyperspectral imaging food fraud’, ‘AI food quality’, and ‘chemometrics adulteration’ as shown in Table 1. The final corpus comprised 83 eligible records published between 2021 and 2026 across 18 peer-reviewed journals. A representative Boolean expression is shown below and the complete strategy adapted to each base with its filters/limits are available in the Supplementary Materials.

### 2.4. Data Extraction

All 83 records were independently double-extracted by two reviewers working in parallel, each blinded to the other’s coding, using the same predefined seven-sheet framework. After independent extraction, the two coding sets were compared field by field, and discrepancies were reconciled by consensus; the agreed values were used for all downstream analyses. Inter-rater reliability was quantified on the six evaluative judgments of Sheet S7 (the fields requiring assessment rather than verbatim transcription). Inter-rater agreement on the six Sheet S7 judgments was high (96.6% overall; Cohen’s κ 0.83–1.00, “almost perfect” by Landis–Koch; PABAK 0.91–1.00), with all 17 discrepancies resolved by consensus.

Data were extracted independently and two reviewers assess the risk of bias of each study and its independence using a structured seven-domain framework operationalized as a seven-sheet Excel workbook: (Sheet S1) Study Information—authors, year, journal, country, food matrix, fraud type, sample size, and funding; (Sheet S2) Instrumental Technology—sensor type, specific instrument, target compounds, sample preparation, preprocessing, and measurement conditions; (Sheet S3) AI Models—algorithm category, specific model, input/output variables, training strategy, cross-validation, external validation, and software; (Sheet S4) Performance Metrics—accuracy, R^2^, RMSE, AUC, F1, sensitivity, specificity, and overfitting discussion; (Sheet S5) Industrial Validation—pilot-scale testing, industrial environment testing, inter-laboratory validation, real supply chain samples, TRL, industrial partners, cost analysis, and scalability; (Sheet S6) Implementation Evidence—routine monitoring application, regulatory integration, adoption barriers, economic constraints, technical expertise required, time-to-result, standard method comparison, and author conclusions; (Sheet S7) Methodological Quality—sample size adequacy, dataset diversity, preprocessing clarity, validation strategy robustness, risk of bias, and overall quality score (1–5).

### 2.5. Methodological Quality Assessment and Its Calibration

Quality was assessed with a five-point structured rubric over six domains: sample-size adequacy (adequate if *n* ≥ 150 total, or justified by design), dataset diversity (geographic, seasonal, and source diversity), preprocessing-description clarity, validation-strategy robustness (external validation weighted most heavily and treated as the pivotal criterion), risk-of-bias categorization (Low/Low–Moderate/Moderate/High), and an overall composite score from 1 (critically limited) to 5 (exemplary). The full decision matrix and modifier flags are provided as Supplementary Table S7. The justification, sensitivity, and potential over-restrictiveness of these thresholds are examined explicitly in Section 4.6. The full classification framework is summarized in Table 2.

### 2.6. Statistical Analysis

To move beyond narrative synthesis, three pre-specified quantitative analyses were conducted on values extracted verbatim from the workbook. (i) A representative classification accuracy per study was taken as the maximum reported classification accuracy in the Performance-Metrics sheet (values constrained to 50–100%); studies reporting only regression metrics were excluded from accuracy comparisons. (ii) Classical-ML versus deep-learning accuracy was compared with the Mann–Whitney U test (two-sided). (iii) The association between high reported accuracy and validation rigor was tested with Fisher’s exact test (accuracy ≥ 99% vs. <99% × robust-validation Y/N) and with Spearman correlation (accuracy vs. quality score). These analyses are descriptive of the literature; because external performance is rarely reported, they characterize reporting patterns rather than true generalization. Statistical analysis was performed using Python 3.14.5.

Formal GRADE certainty rating was not applied, as the review does not synthesize a comparative intervention-effect estimate; its principal outcomes are descriptive characterizations of reporting practice (external-validation frequency, deployment counts). Confidence in the body of evidence is instead conveyed through the six-domain risk-of-bias framework (Table 3), the threshold sensitivity analysis (Section 4.6), and the explicit distinction—maintained throughout Results and Discussion—between interpretation-independent counts and weaker inferential claims.

## 3. Results

### 3.1. Study Selection and General Characteristics

**Figure 1:**
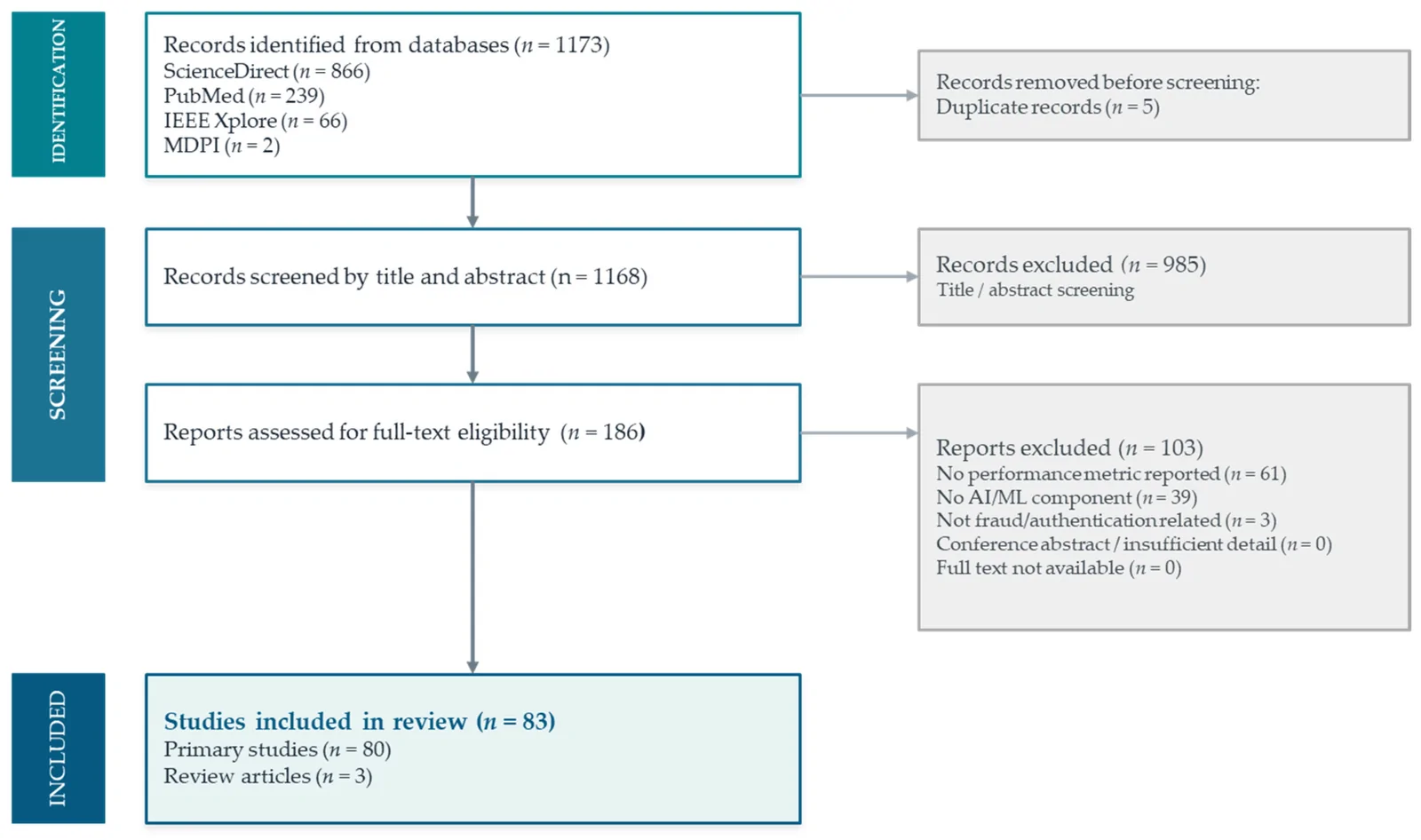
PRISMA 2020 flow diagram.

The final corpus comprised 83 records (See PRISMA diagram, Figure 1) across 18 peer-reviewed journals. The temporal distribution showed marked acceleration: two records in 2021, five in 2022, six in 2023, 18 in 2024, 36 in 2025, and 16 in early 2026. The leading venues were Food Chemistry (18 records, 22%), Journal of Food Composition and Analysis (10, 12%), Food Control (6, 7%), and LWT—Food Science and Technology (6, 7%), reflecting the field’s position at the intersection of analytical chemistry and computational science.

**Figure 2:**
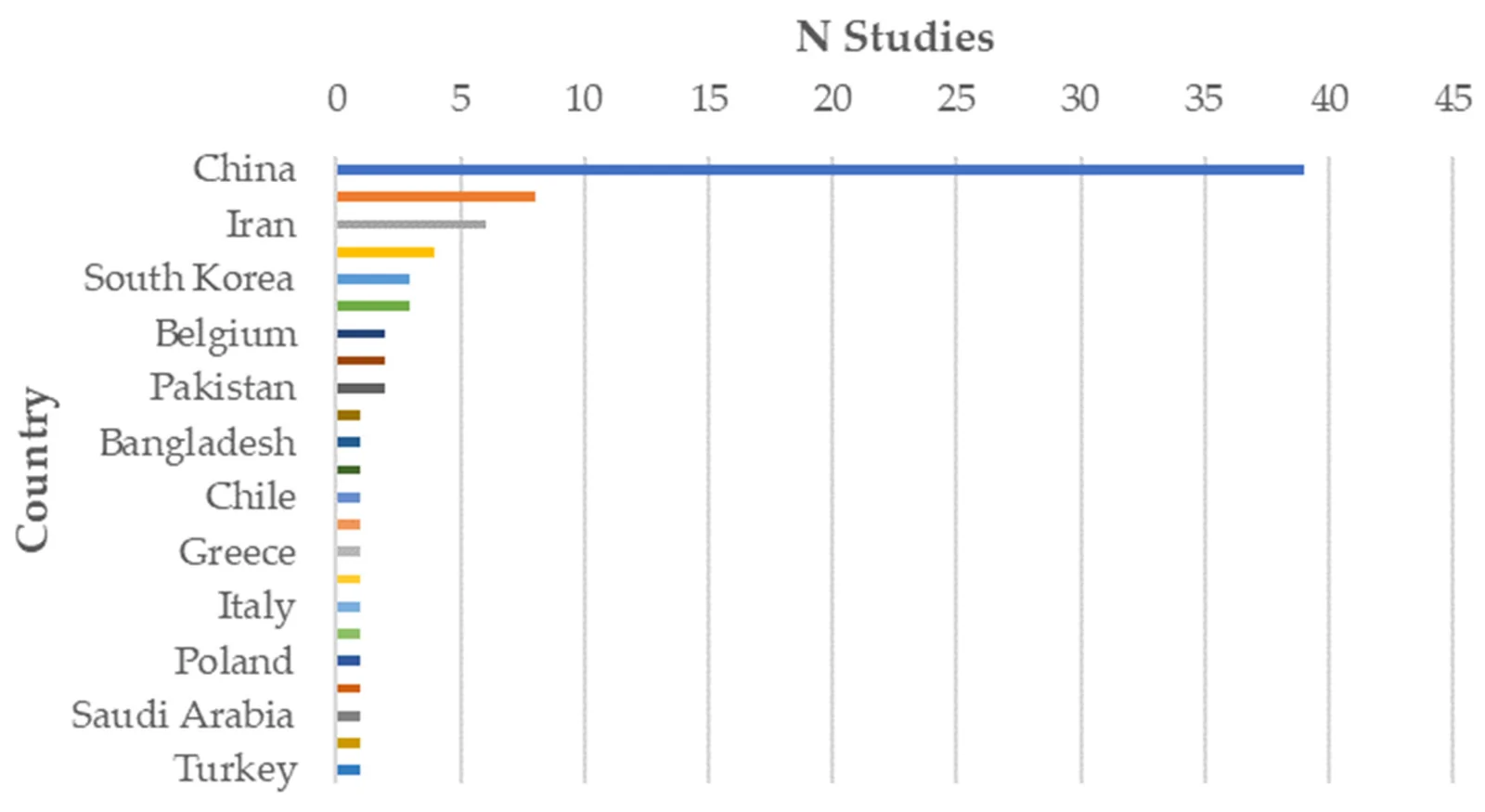
Number of studies per country.

Geographically, China dominated with 38 records (46%), followed by India (8), Iran (6), Spain (4), South Korea (3), and the USA (3), with single-study contributions from many other countries. This concentration is consistent with the broader global distribution of food-science output, but it does raise generalizability questions, since fraud patterns, adulterant availability, and regulatory contexts differ across regions, as shown in Figure 2 and Table 4.

Food matrices were diverse, spanning oils and fats (olive [10,45], camellia [11,24], sesame [8,46], peanut [47], avocado [22], vegetable oils [37,48,49]), dairy (milk [12,15,50,51], milk powder [43,52], tallow [53]), beverages and teas (black/green tea [16,20,21,35], herbal/medicinal teas [31,36], spirits [54], wine [55]), meat and poultry (beef [6,56,57], pork [5,58], goose [4], lamb [42]), spices (turmeric [2,17], saffron [59], black pepper [60], cinnamon [27,61], chilli [3,44]), honey [14,62,63,64,65], rice [66,67,68], and others.

**Table 4:** Summary of key indicators (recomputed from the 83-record workbook).

Indicator	Value	Interpretation
Total records included	83	Comprehensive corpus 2021–2026 (80 primary + 3 reviews)
Studies with NO external validation	82% (68/83)	Predominant validation deficit
Studies with robust/independent validation	18% (15/83)	Minority report a genuinely independent test set
Inter-laboratory validation	0% (0/83)	No ring-trial/regulatory-readiness evidence
Pilot-scale testing	0% (0/83)	No pre-industrial scale-up reported
Industrial-environment testing	1 study [69]	Single in-plant deployment (process monitoring)
Routine-monitoring application	0% (0/83)	No operational deployment documented
Explicit TRL assignment	0 (qualitative discussion in ≈3)	Development stage rarely positioned
Overfitting NOT discussed	51% (42/83)	Half of accuracy claims unqualified by overfitting analysis
Quality score ≥ 4/5	14.5% (12/83)	Most studies of moderate rigor
High risk of bias	23% (19/83)	Substantial minority with limited reliability
Median extractable classification accuracy	≈99–100%	High in-lab performance

Accuracy summary is computed over the subset of studies (*n* = 64) reporting an extractable classification accuracy; regression-only studies are excluded from this table.

### 3.2. Instrumental Technologies

Because many studies combine sensors in data-fusion designs, technologies are reported here as the number of studies in which each platform features (counts therefore overlap). NIR spectroscopy featured in 30/83 studies [2,6,9,10,14,15,16,17,21,23,25,26,27,28,31,35,48,52,55,57,60,63,65,66,70,71,72,73,74] and electronic-nose arrays in 30/83 [1,2,3,4,8,12,21,22,29,30,31,32,33,34,35,36,39,41,58,64,66,69,70,71,72,75,76,77]; gas-chromatography-based systems in 16 [4,11,12,20,22,29,33,39,40,41,42,47,48,69,77,78]; hyperspectral imaging in 13 [9,10,13,16,48,52,57,60,62,73,74,79]; computer-vision/imaging in 15 [18,27,28,43,44,56,57,67,68,71,72,75,76,80,81]; mass spectrometry in seven [7,11,36,41,70,72,82]; FTIR in seven [11,24,37,50,54,55,83]; electrochemical/electronic-tongue in nine [4,32,34,35,36,42,70,72,84]; and UV–Vis/fluorescence [14,45,49] and dielectric/microwave [46,51] platforms in smaller numbers. NIR’s prevalence reflects non-destructive, rapid, low-preparation measurement and the availability of portable instruments suitable for field screening (e.g., the NeoSpectra-class miniaturized sensor used in [17] for turmeric, and the Vis–NIR LED multispectral sensor in [28] for coconut sugar).

A notable trend is the rise of multi-sensor data fusion, which several studies report as outperforming single-sensor baselines. Ren et al. [35] combined NIR, electronic eye, electronic tongue, and electronic nose in a CNN framework, reporting 99.14% on the prediction set; Song et al. [11] reported that mid-level fusion of LA-REIMS lipidomic fingerprints with GC fatty-acid data reached 99.56% classification accuracy for camellia-oil adulteration, exceeding either platform alone.

### 3.3. AI Models and Algorithms

**Figure 3:**
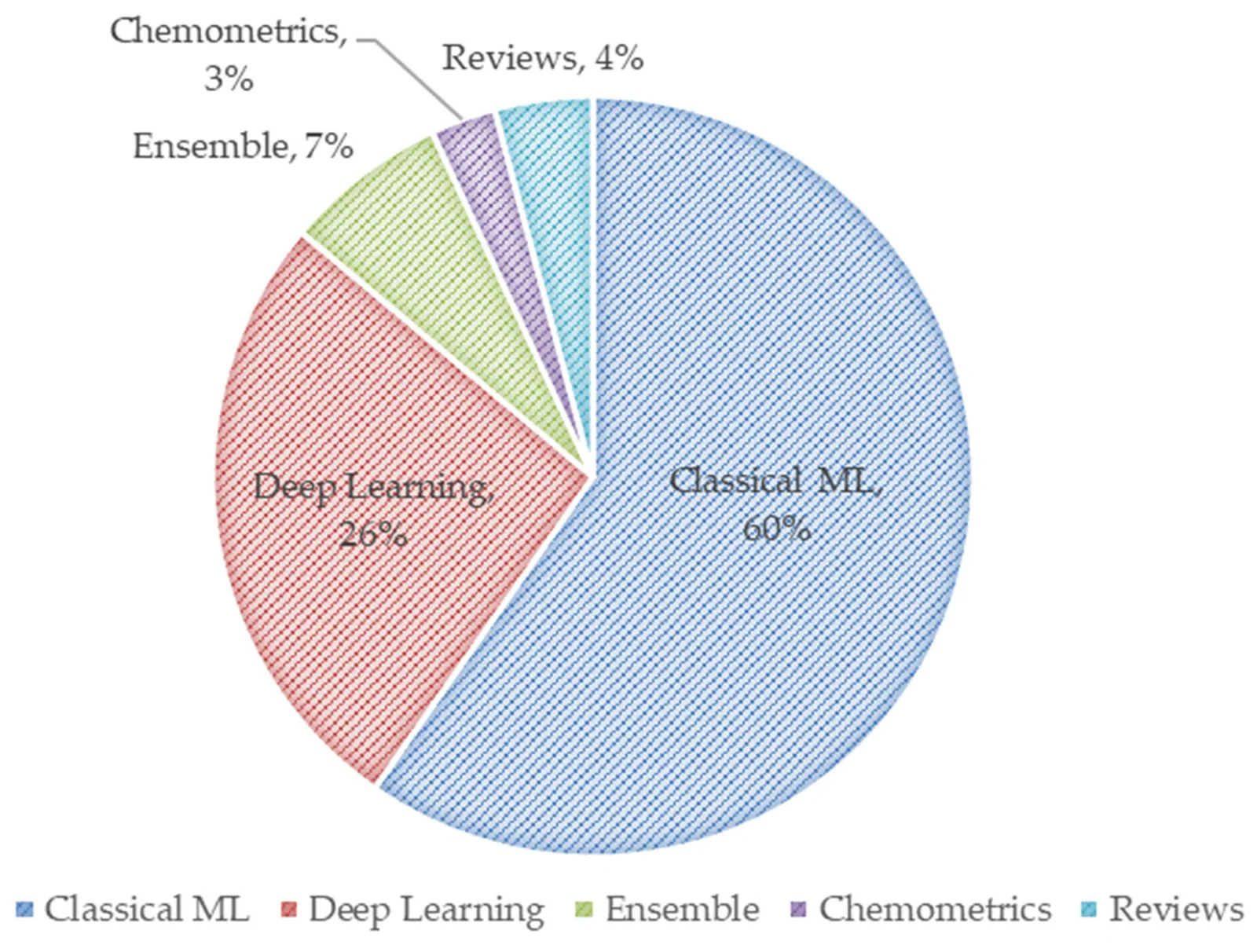
AI algorithm types.

Classical machine learning predominated: 57/83 records were coded as classical-ML designs [3,5,8,12,13,22,25,26,36,41,53,54,56,58,59,65,69,71,84,85], with a further 11 hybrid ML/DL designs [15,35,49,67,80] and 12 deep-learning–only designs [1,4,6,18,30,43,44,57,76,77,79,81]; the three review articles [70,72,78] do not report a primary model. Within classical ML, SVM, Random Forest, and ANN/MLP recur most frequently, often in comparative benchmarking as shown in Figure 3. These methods are well established in chemometrics and remain effective with relatively small datasets—an advantage given the limited size of many authenticated reference collections. Deep-learning designs include CNNs, ResNet/DenseNet variants, and Transformer-based architectures (e.g., the CCST of Shen et al. [42]; the AKCA-Net of Sun et al. [1]).

Training and validation strategies varied: single random train–test splits (commonly 70/30 or 80/20) were the most frequent design, k-fold cross-validation was widely used (often alongside a fixed test set), and only a minority employed a genuinely independent external dataset (Section 3.5). The most rigorous external-validation design in the corpus was the cultivar-disjoint split of Malavi et al. [9], ensuring that no Arabica cultivar appeared in both calibration and external test sets.

#### Statistical Comparison: Classical ML Versus Deep Learning

A common assumption in the field is that deep-learning architectures deliver superior accuracy. The present corpus does not support this assumption. Among studies with an extractable classification accuracy, classical-ML designs (*n* = 49) reported a median of 100.0% and deep-learning designs (*n* = 8) a median of 98.2%; the difference was not statistically significant (Mann–Whitney U = 253.5, *p* = 0.16). Pooling deep learning with hybrid ML/DL designs (*n* = 15, median 99.1%) and comparing against classical ML likewise yielded no significant difference (U = 443.0, *p* = 0.21). Descriptively, classical-ML accuracies were slightly higher on the median and showed marginally greater spread (a small number of classical models at 87–90%), whereas deep-learning accuracies clustered tightly between 90% and 100%.

Two interpretations are consistent with these data and should be weighed jointly. First, on these controlled-laboratory tasks, classical chemometric methods remain at least as accurate as deep learning, while requiring far less data and computation—an argument for parsimony in a field constrained by small authenticated datasets. Second, the absence of a deep-learning advantage may itself reflect a ceiling effect: when median accuracies approach 100% under internal validation, the metric loses the resolution needed to discriminate methods, and any genuine generalization advantage of one family over another would only become visible under external evaluation—which is rarely performed. The non-result is therefore informative about reporting conditions as much as about intrinsic model capability. A counter-example worth noting is [6], in which a deep model (AlexNet) generalized to an external set far better than its classical baselines.

Table 5 describes three panels. Panel A shows that high reported accuracy is broadly distributed across platforms rather than concentrated in any single technology: every major modality has a median ≥ 98.7%, and the four most common platforms (NIR, HSI, electronic nose, imaging) all sit at or near the practical ceiling. Confidence intervals sharpen this picture: the mean accuracy of NIR, HSI, imaging, FTIR, and MS platforms cannot be distinguished from the 100% ceiling (upper bounds at or near 100%), whereas electronic-nose systems are the lone platform whose 95% CI (94.5–98.8%) sits clearly below ceiling and whose dispersion is appreciably larger (CV 4.35% vs. ≤2.6% elsewhere). Formal between-study heterogeneity is extreme by any meta-analytic measure, but—for the reasons detailed in Table 2’s note—this reflects evaluation design and ceiling truncation more than substantively different platform performance, as the non-significant Kruskal–Wallis comparison across platforms confirms (*p* = 0.53). This uniformity is itself diagnostic—when nearly every technology reports near-perfect internal accuracy, the metric provides little basis for choosing among them, and platform selection should instead be driven by deployment factors (cost, portability, throughput, robustness) that the corpus rarely reports. Panel B reinforces this point at the level of model families: the absence of a significant ML–DL difference implies that, on these tasks and under internal validation, architectural sophistication is not the binding constraint on reported performance. Panel C is perhaps the most consequential: external-validation frequency does not rise monotonically over time (0%, 0%, 33%, 6%, 19%, 31%). The apparent 2023 peak rests on only six records and is not stable; pooled across all years, fewer than one in five studies report external validation. The trend line, therefore, offers, at best, weak and noisy evidence of methodological maturation—and certainly not the steady improvement one would expect if the field were converging on deployment-grade evaluation standards.

### 3.4. Performance Metrics: High Internal Precision and Sparse External Evidence

Reported metrics are consistent: classification accuracies almost universally exceed 90%, and many reach 100%. Illustrative examples include Sun et al. ([1], 98.21% ± 0.71% for soybean origin), Lu et al. ([20], 100% F1 for high-grade tea), the turmeric-origin study reaching 100% after SNV preprocessing [2], Tian et al. ([12], 100% for vegetable-oil adulteration in raw milk using a Flash-GC electronic nose with Random Forest), and Firouz et al. ([46], 100% classification with near-unity R^2^ for sesame-oil adulteration using dielectric spectroscopy and ANN).

Crucially, 42/83 studies (51%) do not include an explicit discussion of overfitting [1,2,3,7,8,15,20,24,26,27,28,34,35,36,37,38,41,44,45,46,47,48,49,51,52,53,54,55,56,59,60,64,68,73,74,75,77,80,82,84,85], even where reported accuracies approach the measurement ceiling. Where overfitting is addressed (41 studies; e.g., [4,5,6,9,10,11,12,13,14,16,17,18,21,22,23,29,30,31,33,39,40,43,50,57,58,62,63,65,66,67,69,71,78,79,81,83], it is typically handled through cross-validation, regularization, or permutation tests; genuinely independent external validation on novel samples from different laboratories or supply-chain positions remains infrequent. Several studies do, however, document train–test gaps within their own design—for example, Su et al. [47] reported a regression R^2^ of 0.9153 (train) versus 0.7254 (test), and Song et al. [11] reported 99.56% on the internal test set versus 97.80% on a 500-sample real-time blind validation in the LiveID™ system.

#### Direct Test: Does High Reported Accuracy Imply Overfitting?

A recurring critique—implicit in much of the literature and in earlier drafts of this review—is that very high accuracy is itself a signature of overfitting. We tested this proposition directly rather than asserting it. If high accuracy were primarily an artifact of overfitting, one would expect high-accuracy studies to be disproportionately associated with weak validation and lower methodological quality. The data shows:

- Accuracy × validation rigor. Among studies reporting ≥99% accuracy, 23.70% had robust external validation, versus 14.30% among studies reporting <99% (Fisher’s exact test, *p* = 0.51). The association is not significant and, if anything, runs opposite to the overfitting hypothesis.
- Accuracy × quality score. Reported accuracy did not decline with methodological-quality score; the correlation was weakly positive and non-significant (Spearman ρ = 0.15, *p* = 0.23). Mean accuracy was 97.50% (score 2), 98.30% (score 3), 99.60% (score 4), and 100% (the single score-5 study).
- Externally validated vs. internal-only. Studies with robust external validation did not report lower accuracy than internal-only studies (median 100.0% vs. 99.60%; Mann–Whitney *p* = 0.45).

These results caution against the simple equation, high accuracy = overfitting. Three readings are compatible with the evidence, and we present them without privileging the one most convenient for the review’s thesis. (a) The hypothesis may be false for much of this corpus: classical chemometric tasks on well-separated chemical classes can be genuinely easy, so near-perfect internal accuracy need not signal overfitting. (b) The test may be underpowered to detect the effect: external performance is reported in too few studies (15/83) for the comparison to be sensitive, and the within-study gaps that are reported (e.g.,: ΔR^2^ ≈ 0.19; logistic baseline: ≈99% internal vs. ≈69% external) show that substantial generalization loss does occur in specific cases. (c) Overfitting may be masked by the very practice the review critiques—without external evaluation, an overfit model and a genuinely generalizable model can report identical internal accuracy, so the metric cannot, even in principle, separate them. Reading (c) is the most defensible synthesis: the problem is not that high accuracy proves overfitting, but that the dominant evaluation design makes overfitting undetectable. This reframing is more precise than the original claim and shifts the argument from a contestable empirical assertion to a statement about evidential adequacy.

Proposed direct test for future studies. To convert this from an inference to a measurement, we recommend a standardized protocol that every study could report: train the model under internal validation and record P_internal, and then evaluate the frozen model on an independently collected set (different instrument, operator, batch, or laboratory) and record P_external. The generalization gap ΔG = P_internal − P_external would then be reported alongside headline accuracy. A field-level prediction follows: if high internal accuracy is benign, ΔG should remain small and uncorrelated with internal accuracy; if it reflects overfitting, ΔG should grow with internal accuracy and with model complexity relative to sample size. Because ΔG is currently observable in only a handful of studies, the hypothesis remains, at present, neither confirmed nor refuted—an honest position that the corpus, as it stands, requires.

### 3.5. External Validation: A Predominant but Not Universal Absence

Sixty-eight of 83 studies (82%) reported no external validation of any kind. Among the 15 studies that did [5,6,7,9,10,11,12,18,23,25,39,40,43,57,68], rigor varied: some used held-out concentration ranges from the same laboratory batch (e.g., [23]), supplementary conditions from the same design (e.g., [39,40]), or real-time blind tests on samples prepared by the same team (e.g., [11,18]). A smaller subset—[6,9,10,12,43,68], and [7]—employed validation that can be characterized as genuinely independent (independent commercial samples, cultivar-disjoint splits, or multi-origin/multi-cut external sets). The absence of inter-laboratory validation, by contrast, was absolute (0/83): no study validated across multiple laboratories using independently calibrated instruments. Measured against AOAC and [86] method-validation expectations, none of the reviewed systems would currently meet the standard required for accredited routine implementation. We note this as an empirical observation about the corpus rather than as proof that any individual method would fail such validation if it were attempted, as presented in Table 6.

### 3.6. Industrial Validation and Implementation Evidence

The detection–prevention gap is most visible in deployment data. No study reported pilot-scale testing; one study ([69], Kan et al.) tested in a genuine industrial environment, using PTR-TOF-MS with machine learning to classify the fermentation stage of soy-sauce production across three 100 tonnes industrial fermenters (QDA 100%; Extra Trees 99.83%). This is a process-monitoring application rather than fraud detection, but it shares instrumental platforms, model families, and validation challenges with fraud detection, illustrating a plausible knowledge-transfer pathway. No study documented routine-monitoring application in an operational context, and no inter-laboratory validation was identified.

Explicit Technology Readiness Levels were not assigned in any study; a small number discussed deployment engineering qualitatively (e.g., Song et al. [11] describe factory installation of LA-REIMS as ‘challenging yet feasible’, requiring dedicated computing infrastructure, robotic handling, and automated pipelines; Zhu et al. [57] reports a working sub-USD-60 prototype; Machuca et al. [62] notes future real-time implementation). Cost analysis was provided in 14 studies [11,12,22,27,28,29,30,38,44,45,49,50,57,82] and scalability discussed in 69, though usually at the level of aspiration rather than quantified techno-economic assessment.

Regulatory integration was mentioned in 20 studies (24%) [2,7,10,12,17,18,22,34,43,50,62,63,65,66,68,69,70,79,81,83], typically by reference to existing frameworks (Codex Alimentarius, EU directives, national standards) as motivation. The most concrete regulatory linkage was Tian et al. [12], who validated against ISO 17678:2019 and confirmed one genuine positive among 300 real commercial milk samples. No study reported formal adoption or integration of its method into a regulatory enforcement workflow.

### 3.7. Methodological Quality Assessment

Quality was predominantly moderate. Forty-nine studies (59%) scored 3/5, 22 (27%) scored 2/5 [4,8,16,26,27,32,33,34,36,42,52,53,54,55,60,62,65,69,75,77,79,84], 11 (13%) scored 4/5 [6,7,10,11,12,18,25,31,43,57,68], and a single study—Malavi et al. [9], a NIR-HSI study of Robusta-in-Arabica coffee adulteration with cultivar-disjoint external validation—achieved 5/5. Risk of bias was Moderate in 49 studies (59%), High in 19 (23%) [4,8,16,27,32,33,34,42,52,53,54,55,60,62,65,75,77,79,84], Low or Low-to-Moderate in 11 (13%), and Not Applicable (reviews [70,72,78], plus one further review-type record) in four. Among the six quality domains, preprocessing was well described in most studies (73/80 non-review records), but dataset diversity was adequate in only 28/80 and robust validation in only 15/80, with sample size adequate in 44/80. The binding constraints on quality were therefore validation rigor and dataset diversity rather than preprocessing transparency.

## 4. Discussion

### 4.1. The Detection–Prevention Gap: What the Evidence Does and Does Not Establish

The corpus supports a well-evidenced central observation: AI-assisted instrumental systems demonstrate high analytical accuracy under controlled conditions, while the evidence that they prevent food fraud in real supply chains is minimal. This conclusion rests on hard, interpretation-independent counts: 0/83 inter-laboratory validations, 0/83 routine-monitoring deployments, 0/83 pilot-scale tests, and a single in-plant (process-monitoring) study. These are facts about the literature, not inferences, and they justify the claim that detection capability has substantially outpaced demonstrated prevention capability.

We are more cautious about the stronger causal claim that the gap is ‘primarily systemic rather than technological’. The limitation-coding in Table 6 shows systemic limitations outnumbering technological ones (161 vs. 100 coded statements), which is consistent with that interpretation. But coded limitation counts partly reflect what authors choose to disclose, and the same data are compatible with a more measured reading: the field is early in its translational trajectory, and the absence of deployment evidence may reflect the normal lag between proof-of-concept and operationalization as much as a misaligned incentive structure. Both readings are defensible; the data adjudicate the descriptive claim (the gap exists) far more firmly than the causal one (why it exists).

### 4.2. The Validation Deficit and Its Implications

The 82% rate of absent external validation is the most consequential methodological feature of the corpus. Without external validation, reported accuracies cannot be reliably extrapolated to deployment conditions that include seasonal compositional variation, different origins and processing, different operators, and different instrument calibration states. The most rigorous studies—[6,9,10,12], and [7]—share a substantial investment in validation design: cultivar-disjoint splits [9], multi-cut/multi-country external sets [6], validation against a regulatory reference method on 300 commercial samples [12], and independent commercial test samples in a proteomics workflow [7]. Their existence demonstrates that rigorous external validation is achievable within current resource constraints; their rarity indicates that it is not yet the norm. Importantly, these well-validated studies still report high accuracy, which tempers—without eliminating—concern that internal accuracies are uniformly inflated.

### 4.3. Technology Concentration and Generalizability

The concentration of research in China (46%) and the dominance of NIR and electronic-nose platforms create specific generalizability considerations. Models calibrated on regionally specific matrices (e.g., Yunnan black tea [35]; Jinhua ham [33]; Shanxi vinegar) encode reference databases and threshold calibrations that are not directly transferable to other regulatory and commercial contexts without revalidation. Certain fraud modalities—especially species substitution detectable mainly through DNA or proteomic methods—remain underrepresented; the proteomics study of Venegas et al. [7], which detected walnut adulteration at 1% *w*/*w* on independent commercial samples, illustrates a methodological direction that is largely absent from the broader literature.

### 4.4. Reporting Conventions and the Interpretation of High Accuracy

A recurrent rhetorical pattern describes methods as ‘promising’, ‘efficient’, or ‘feasible’ for ‘real-time on-site detection’, while the evidence base is laboratory-scale with internal validation only. The statistical analyses in Section 3.6 refine how this pattern should be criticized. The legitimate concern is not that a 100% accuracy on a small dataset necessarily indicates an overfit model, but that the prevailing evaluation design cannot distinguish an overfit model from a generalizable one. A Random Forest reporting 100% on 72 samples from three provinces [2], or a dielectric-spectroscopy ANN reporting 100% with near-unity R^2^ on 135 oil samples from one laboratory [46], is best described not as ‘proven to be overfit’ but as ‘not yet shown to generalize’. This distinction matters for fair appraisal of the literature and for the design of future studies.

### 4.5. What Would Genuine Translation Look Like?

Bridging the gap would require: (1) external validation using genuinely independent sources (different laboratories, operators, origins, seasons); (2) inter-laboratory ring-trial validation comparable to regulatory method-adoption standards; (3) inclusion of real commercial supply-chain samples rather than exclusively laboratory blends; (4) techno-economic analysis covering instrument, consumable, training, and throughput costs; and (5) explicit Technology Readiness Level assignment positioning each study on the path from concept (TRL 1) to operational deployment (TRL 9). Song et al. [11] come closest, combining high internal accuracy (99.56%) with a 500-sample real-time blind test (97.80%) and explicit discussion of factory-installation engineering—the kind of translational evidence the broader literature needs.

### 4.6. Are the Quality Criteria Too Restrictive? Threshold Justification and Sensitivity Analysis

Because the quality framework strongly shapes the headline conclusions, its thresholds deserve explicit scrutiny. We address four questions directly, using the workbook to test the framework against itself.

(i) Could the criteria be too restrictive in some cases? The decision matrix is deliberately conservative: robust (independent) validation is the pivotal criterion, and its absence prevents a low-risk classification. This is defensible for a review whose question is about real-world prevention, where independent validation is the single most informative signal of deployability. However, the framework does not collapse to a single criterion. Empirically, all 19 high-risk studies failed both dataset-diversity and validation criteria simultaneously; none was rated high-risk on the basis of one failing field alone. This indicates that the rubric is not mechanically over-penalizing—high risk requires converging deficiencies, not a single threshold miss.
(ii) Does the configuration penalize domains with naturally small datasets? This is the most important fairness concern, since proteomics, NMR, and certain spectroscopic authentication tasks are intrinsically low-n. The workbook shows the rubric already accommodates this. The clearest example is Venegas et al. [7]: with only 27 references + 8 independent commercial samples (sample size flagged inadequate against the *n* ≥ 150 threshold), the study still scored 4/5 (Low risk) because it satisfied dataset diversity and, critically, genuine external validation. Conversely, small-n studies that scored 2/5 (e.g., [56], tallow NMR, *n* = 28; [36], medicinal tea, *n* = 36; [78], soy sauce, *n* = 54) failed not because of n alone but because they also lacked diversity and independent validation and were exploratory in design. In other words, a small sample size is necessary but not sufficient for a low score; the rubric rewards small-but-rigorous studies that compensate with diversity and external testing.
(iii) Why were these parameters/thresholds chosen? The *n* ≥ 150 threshold approximates the lower bound at which high-dimensional spectral models can be evaluated with a meaningful hold-out partition while retaining class balance; it is a heuristic, not a hard scientific constant, and the rubric explicitly permits intermediate *n* (50–149) to pass when class-balanced and dimensionality-reduced. Independent validation was given pivotal weight because it is the criterion most directly tied to the review’s prevention question. Preprocessing clarity and dataset diversity were included because they govern reproducibility and generalizability, respectively. These choices privilege deployability over in-lab optimization—an intentional alignment with the research question, which we state openly so readers can recalibrate if their interest is purely methodological.
(iv) Were alternative scenarios tested, and how would results change under a less restrictive validation criterion? Yes. We re-scored the corpus under a relaxed definition that counts partial/quasi-external validation (e.g., held-out concentration ranges or same-team blind tests) as ‘robust’. Under this relaxation, the count of studies meeting the validation criterion rises modestly from 15 to 20 (18% → 24%), and the headline statement changes only in degree, not kind: a clear majority (≈76%) of studies would still lack robust validation. Notably, the high-risk tier is essentially unaffected, because every high-risk study fails on dataset diversity as well as validation—so loosening only the validation rule does not rescue them. The inter-laboratory finding (0/83) is invariant to any threshold choice as presented in Table 7, since it is a simple presence/absence count. The central conclusions are therefore robust to reasonable relaxation of the most contested threshold; what relaxation buys is a softer characterization of the degree of the deficit, not its existence.

### 4.7. Limitations of This Review

Several limitations apply. The corpus of 83 records, while comprehensive for 2021–2026, may under-represent the gray literature, conference proceedings, and non-English studies. Publication bias may favor positive results, potentially inflating the typical reported performance. Accuracy figures were extracted as the maximum reported classification accuracy per study; this is a conservative, comparable proxy but discards within-study distributional detail and excludes regression-only studies, so the ML-vs-DL and accuracy-association analyses should be read as characterizations of reporting patterns rather than definitive performance estimates. Model-family coding (ML/DL/hybrid) involves judgment for studies that benchmark many algorithms. Methodological-quality scoring, though criterion-based, involves boundary judgments that another assessor might resolve differently; the sensitivity analysis in Section 4.6 partially addresses this. Finally, the rapid publication pace means some early-2026 studies may be incompletely indexed.

## 5. Conclusions

This systematic review of 83 records (2021–2026) yields three empirically grounded conclusions, stated at the level of confidence the evidence supports.

First, internal analytical precision is consistently high and architecture-agnostic. AI-assisted instrumental systems routinely achieve classification accuracies above 95% and regression R^2^ above 0.90 under laboratory conditions, and classical ML performs at least as well as deep learning on these tasks (no significant difference; *p* = 0.16). This reflects a methodologically mature analytical foundation and argues for parsimonious model choice given small authenticated datasets.

Second, external evidence is sparse and does not show clear temporal improvement. Sixty-eight of 83 studies (82%) reported no external validation; none achieved inter-laboratory validation; none documented routine monitoring; and external-validation frequency over time is noisy rather than rising. We deliberately do not claim that high internal accuracy proves overfitting—a hypothesis the corpus could neither confirm nor refute (Section 3.4)—but rather that the prevailing evaluation design leaves generalization, and therefore overfitting, undetectable.

Third, a measurable detection–prevention gap exists, strongly evidenced by deployment counts and weakly explained by the available data. The literature has shown that AI systems can detect adulteration under controlled conditions; it has not yet shown that they prevent fraud in real supply chains. Whether this gap is ‘primarily systemic’ is plausible but not established to the same standard as the gap itself.

Author motivations and constructive directions. Reading the authors’ own stated motivations across the corpus is illuminating and largely convergent. Recurrent drivers include: protecting high-value commodities and consumer health (e.g., toxic-adulterant detection in mustard [80]; allergen-relevant walnut adulteration [7]); enabling rapid, non-destructive, low-cost on-site screening as an alternative to slow reference methods (the dominant rationale behind portable-NIR and electronic-nose studies, e.g., [15,17,77]); supporting regulatory compliance against named standards (ISO 17678:2019 in [12]; Codex frameworks in olive- and avocado-oil studies [10,22]); and improving process control with direct industrial relevance (in-plant fermentation monitoring [69]; factory-deployment engineering [11]). These motivations are socially valuable; the gap this review identifies is not a failure of intent but of evaluation design. The most translation-oriented authors in the corpus [6,7,9,11] already model the path forward—independent validation, real commercial samples, and explicit deployment discussion—demonstrating that the required reorientation is achievable within current incentives rather than requiring their wholesale replacement.

Closing the gap will require a shift in success criteria: from maximizing classification performance under laboratory conditions toward demonstrating measurable, externally validated impact within real food systems. Concretely, the field would benefit from routine reporting of the generalization gap ΔG, inter-laboratory ring trials, inclusion of commercial supply-chain samples, techno-economic analysis, and TRL transparency. Until these become standard, the impressive analytical performance documented here is best understood as a demonstration of what is technically possible—a necessary precondition for, but not yet evidence of, prevention.

## Figures and Tables

**Table 1:** General and specific Boolean equation.

General search: (“electronic nose” OR “electronic tongue” OR spectroscopy OR “hyperspectral imaging”) AND (“artificial intelligence” OR “machine learning”) AND (“food fraud” OR adulteration). Specific search: ((“food fraud” OR “food adulteration” OR “food authentication” OR “adulteration detection”) AND (“machine learning” OR “deep learning” OR “artificial intelligence” OR “neural network” OR chemometrics OR “support vector machine” OR “random forest” OR “convolutional neural network”) AND (NIR OR “near-infrared” OR “electronic nose” OR hyperspectral OR spectroscopy OR “gas chromatography” OR Raman OR FTIR)) NOT (“review” OR “survey” OR “meta-analysis”). Note: the explicit NOT (“review”) clause was used for the specific-search strand; three review articles meeting the meta-level inclusion criterion were nonetheless retained for the broader synthesis.

**Table 2:** Risk of bias classification framework.

**Risk of Bias Classification Framework—Methodological Quality**			
**Part A—Field-Level Y/N Criteria**			
**Field**	**Criterion for Y (Assign Y When…)**	**Criterion for N (Assign N when…)**	**Notes/Edge Cases**
Sample Size Adequate	*n* ≥ 150 total samples (train + test combined) Intermediate (50–149): Y only if class-balanced AND dimensionality reduction is applied	*n* < 50 → automatic N *n* = 50–149 with high-dimensional spectral data and NO dimensionality reduction → N	Sum train + test splits before applying threshold. State the exact n used.
Dataset Diversity Adequate	Samples include ≥2 of: Multiple geographic origins Multiple producers/suppliers Multiple harvest seasons or lots Commercially sourced market samples	All samples from a single producer, lot, or geographic origin OR dataset is 100% lab-spiked with no real commercial samples	Lab-spiked only = N regardless of n. Note in ‘Notes’ column of Sheet 7.
Clear Preprocessing Description	Paper explicitly states: Which preprocessing steps were applied (e.g., SNV, MSC, derivative) Order of application Parameters used (window size, polynomial order, etc.)	Preprocessing mentioned only generically (e.g., ‘data were preprocessed’) OR omitted entirely OR impossible to reproduce from description	Reproducibility test: could another lab replicate the preprocessing exactly?
Validation Strategy Robust	Y ONLY if an independent dataset is used—collected at a different time, different instrument, OR different laboratory. Must be explicitly declared in the paper.	Validation relies solely on: k-fold cross-validation (any k) Leave-one-out CV Random train/test split from the same dataset → All of these = N regardless of accuracy reported	PIVOTAL CRITERION: Its absence prevents Low risk and elevates classification by ≥1 level.
**Part B—Risk of Bias: Decision Matrix**			
**Risk Level**	**Conditions (ALL must be met unless OR stated)**		**Score range**
Low	Sample Size Y AND Dataset Diversity Y AND Clear Preprocessing Y AND Validation Robust Y → All four fields must be Y. Any single N disqualifies Low risk.		4–5
Moderate	Validation Robust Y AND at least 2 of remaining 3 fields are Y —OR— Validation Robust N BUT all other 3 fields Y AND *n* ≥ 150 (Robust validation is the pivotal criterion—its absence always elevates risk ≥1 level)		3
High	Validation Robust N AND ≥2 of remaining fields are N —OR— *n* < 50 regardless of other fields —OR— Dataset is entirely lab-spiked AND Validation Robust N		1–2
**Part C—Overall Quality Score (1–5)**			
**Score**	**Description**	**Implied Risk Level**	**Typical profile**
5	Exemplary rigor	Low	External validation + diverse dataset + full preprocessing disclosure
4	Good design; minor limitations	Low–Moderate	Internal CV + large *n* + commercial samples + clear preprocessing
3	Acceptable with notable gaps	Moderate	Cross-validation only, adequate n, one major gap
2	Major limitations; limited generalizability	High	Small n, spiked only, no external validation
1	Critical flaws; results unreliable	High	n < 50, no preprocessing description, no validation strategy
**Part D—Modifier Flags (elevate risk level when present)**			
**Flag**	**Condition**	**Effect on classification**	**Action required**
Artificial dataset	Adulterants added in controlled proportions in lab; no market samples included	Elevate 1 level (e.g., Moderate → High)	Note ‘artificial dataset’. Notes column
Overfitting not discussed	Accuracy >95% reported with NO mention of regularization, generalization risk, or overfitting	Elevate 1 level	Mark Overfitting Discussed = N.
Very small classes	Any class in the classification task has <10 samples	Elevate 1 level	Record actual class sizes.
Industry-funded, no disclosure	Private company funding with commercial interest AND no conflict-of-interest statement	Flag only—do NOT auto-elevate	Note funding source. Funding column; flag for reviewer attention
**Part E—Decision Flowchart (Step-by-Step Classifier)**			
Apply the steps below in order for each study. Stop at the first condition that matches.			
**STEP 1|Check Sample Size**			
Question	Is *n* < 50?		
Yes path	YES → HIGH risk (Score 1–2). Stop here.		
No path	NO → Proceed to Step 2		
**STEP 2|Check Dataset Diversity**			
Question	Is the dataset 100% lab-spiked with no real market samples?		
YES path	YES → Flag ‘artificial dataset’. Proceed but apply modifier (+1 level at end).		
NO path	NO → Proceed to Step 3		
**STEP 3|Check Validation Strategy**			
Question	Is there a truly independent external dataset (different time/instrument/lab)?		
Yes path	NO → Cannot be Low risk. Proceed to Step 4 for Moderate vs. High.		
No path	YES → Proceed to Step 4		
**STEP 4|Count Y Fields**			
Question	How many of the 4 Y/N fields are Y? (Sample Size, Diversity, Preprocessing, Validation)		
Yes path	All 4 = Y → Low risk (Score 4–5)		
No path	3 Y (incl. Validation) → MODERATE (Score 3) 2 Y or fewer → HIGH (Score 1–2)		
**STEP 5|Apply Modifier Flags**			
Question	Are any modifier flags present? (artificial dataset/overfitting undisclosed/tiny classes)		
Yes path	YES → Elevate final risk level by 1 (Low → Moderate, Moderate → High). Cannot exceed High.		
No path	NO → Final classification stands		
**Final Output → Enter Risk of Bias (Low/Moderate/High) and Quality Score (1–5)**			

**Table 3:** Field-level criteria of the risk-of-bias framework (condensed). Decision matrix: Low = all four Y; Moderate = robust validation plus ≥2 of remaining Y, or strong design with *n* ≥ 150 but validation N; High = validation N with ≥2 remaining N, or *n* < 50.

Field	Assign Y When…	Assign N When…
Sample size adequate	*n* ≥ 150 (train + test); or 50–149 if class-balanced with dimensionality reduction	*n* < 50; or 50–149 high-dimensional spectral data with no dimensionality reduction
Dataset diversity adequate	≥2 of: multiple origins/producers/seasons-lots/commercial market samples	single producer, lot, or origin; or 100% lab-spiked with no commercial samples
Clear preprocessing	steps, order, and parameters explicitly stated and reproducible	generic mention only, omitted, or not reproducible
Validation robust	independent dataset (different time, instrument, or laboratory), explicitly declared	only k-fold CV, leave-one-out, or random split from the same dataset

**Table 5:** Cross-cutting quantitative analyses derived from the workbook.

**Panel A—Reported Classification Accuracy by Primary Analytical Technology**							
**Primary Technology**	***n***	**Median (%)**	**Mean (%)**	**95% CI (Mean) †**	**SD**	**Range (%)**	**CV (%) ‡**
NIR spectroscopy	15	100.0	99.2	98.2–99.9	1.73	94–100	1.75
Hyperspectral imaging (HSI)	11	100.0	98.9	97.6–99.8	2.13	93–100	2.15
Electronic nose	14	98.7	96.8	94.5–98.8	4.21	87–100	4.35
Imaging/computer vision	7	100.0	99.2	98.3–99.9	1.17	97–100	1.18
GC-based systems	5	99.6	98.5	96.2–99.9	2.56	94–100	2.59
FTIR	4	100.0	99.4	98.1–100.0	1.30	97–100	1.31
Mass spectrometry	2	100.0	100.0	n/a (k = 2)	0.00	100–100	0.00
**Panel B—Classical ML vs. Deep Learning (reported accuracy)**							
**Model family**	***n***		**Median (%)**		**Mean (%)**		**Range (%)**
Classical ML	49		100.0		98.4		87–100
Deep learning (only)	8		98.2		97.0		90–100
Hybrid ML/DL	7		99.1		98.6		95–100
ML vs. DL test	—		Mann–Whitney U = 253.5		*p* = 0.16		
**Panel C—External-validation frequency over time**							
Year	2021		2022		2023		2024
Records (n)	2		5		6		18
With external validation	0		0		2		1
% with external validation	0%		0%		33%		6%
Year	2025		2026		All years		
Records (n)	36		16		83		
With external validation	7		5		15		
% with external validation	19%		31%		18%		

† 95% confidence intervals for the mean were estimated by percentile bootstrap (10,000 resamples) and bounded at 100%, because reported accuracy is ceiling-limited and left-skewed; normal-theory t-intervals would exceed 100% and are not shown. The MS interval is omitted (k = 2). ‡ CV = coefficient of variation (SD/mean × 100), reported here as a descriptive between-study heterogeneity indicator alongside SD and range. Heterogeneity. Accuracy dispersion is low for most platforms (CV ≤ 2.6%) but markedly higher for electronic-nose systems (CV 4.35%; range 87–100%), the only modality whose 95% CI lies clearly below 100%. A Kruskal–Wallis test across the five technologies with k ≥ 3 found no significant difference in accuracy distribution (H = 4.17, *p* = 0.53), consistent with a ceiling effect rather than genuine platform superiority. A supplementary random-effects proportion meta-analysis (Freeman–Tukey double-arcsine, DerSimonian–Laird), treating each study’s maximum reported accuracy as a proportion over its total reported sample size, returned extreme statistical heterogeneity (overall Cochran Q = 4174.6, df = 61, *p* < 0.001; I^2^ = 98.5%; τ^2^ = 0.018; per-technology I^2^ from 0% [FTIR] to 99.5% [HSI]). These formal values are almost certainly inflated and are reported for completeness only: primary studies rarely disclose the test-set denominator (so total n was used, overstating precision), ‘maximum reported accuracy’ is an optimistic point estimate rather than a fixed-denominator proportion, and internal-only evaluation conflates true heterogeneity with differences in task difficulty. Formal I^2^/τ^2^ should therefore not be read as classical meta-analytic heterogeneity of a common effect; the descriptive CI, SD, range, and CV are the more defensible summaries. Primary technology assigned per study by dominant platform; data-fusion studies allocated to the leading modality. Counts cover studies with an extractable accuracy value. Model-family rosters—classical ML (57), deep-learning-only (12), hybrid ML/DL (11). The accuracy test uses the subset of each roster with an extractable classification accuracy.

**Table 6:** Distribution of limitations by category (primary classification).

Category	Code	Count	% of Total	Affected Studies	Interpretation
Technological	T	100	36%	62 (≈75%)	Instrument/algorithm limits—often partially solvable
Systemic	S	161	59%	70 (≈84%)	Design/organization limits—dominate the corpus
Regulatory/Normative	R	5	2%	5 (≈6%)	Weak integration with application frameworks
Incentive/Reporting	I	9	3%	11 (≈13%)	Metrics prioritized over translation
Total	—	275	100%	—	—

Counts reflect coded limitation statements; a single study may contribute multiple limitations across categories. Percentages of affected studies are expressed relative to 83 records. The predominance of systemic over technological limitations is consistent with—but does not by itself prove—the review’s interpretation that the principal constraint is organizational rather than technical.

**Table 7:** Sensitivity of validation findings to threshold choice.

Criterion/Scenario	Strict Rubric	Relaxed Rubric
Studies meeting robust-validation criterion	15 (18%)	20 (24%)
Studies lacking robust validation	68 (82%)	63 (76%)
High-risk-of-bias studies	19 (23%)	19 (23%)
Inter-laboratory validation	0 (0%)	0 (0%)

Relaxed rubric counts partial/quasi-external validation as meeting the validation criterion. The high-risk and inter-laboratory rows are unchanged because they do not depend on the relaxed criterion in isolation.

## Data Availability

The review protocol is publicly available in Zenodo at DOI: 10.5281/zenodo.20785718. All data analyzed during this study were extracted from published peer-reviewed articles included in the reference list.

## Supplementary Materials

The following supporting information can be downloaded at: https://www.mdpi.com/article/10.3390/foods15183185/s1.
