# Supplementary material for: AI-Driven Food Fraud Detection Systems: A Critical Systematic Review of the Detection–Prevention Gap
Source: Foods. 2026 Sep 9;15(18):3185. doi: 10.3390/foods15183185 (PMC13605032; doi:10.3390/foods15183185)
Supplement: Supplementary file 1 [file foods-15-03185-s001.zip › PRISMA checklist.pdf]

PRISMA 2020 Checklist

AI-Driven Food Fraud Detection Systems: A Critical Systematic Review of the Detection–Prevention Gap:

Section and Topic	Item #	Checklist item	Location where item is reported
**TITLE**			
**Title**	**1**	Identify the report as a systematic review.	**OK**<br>Title: "…A Critical Systematic Review of the Detection–Prevention Gap".
**ABSTRACT**			
**Abstract**	**2**	See the PRISMA 2020 for Abstracts checklist.	**OK**<br>Abstract
**INTRODUCTION**			
**Rationale**	**3**	Describe the rationale for the review in the context of existing knowledge.	**OK**<br>1.1 (The Persistent Problem of Food Fraud) and 1.2 (The Rise of AI-Assisted Detection).
**Objectives**	**4**	Provide an explicit statement of the objective(s) or question(s) the review addresses.	**OK**<br>1.3 and 1.5
**METHODS**			
**Eligibility criteria**	**5**	Specify the inclusion and exclusion criteria for the review and how studies were grouped for the syntheses.	**OK**<br>2.2 (Eligibility Criteria)
**Information sources**	**6**	Specify all databases, registers, websites, organisations, reference lists and other sources searched or consulted to identify studies. Specify the date when each source was last searched or consulted.	**OK**<br>2.3
**Search strategy**	**7**	Present the full search strategies for all databases, registers and websites, including any filters and limits used.	**OK**<br>2.3 and boolean ecuactions are available in supplementary material.
**Selection process**	**8**	Specify the methods used to decide whether a study met the inclusion criteria, including how many reviewers screened each record and each report retrieved, whether they worked independently, and if applicable, details of automation tools used.	**OK**<br>2.3–2.4
**Data collection process**	**9**	Specify the methods used to collect data from reports, including how many reviewers collected data, whether independent, processes for confirming data, and automation tools.	**OK**<br>2.4 (Data Extraction)
**Data items**	**10a**	List and define all outcomes for which data were sought. Specify whether all compatible results were sought, and if not, the methods used to decide which results to collect.	**OK**<br>2.4 (Sheet 4: Performance Metrics) and 2.6 (maximum accuracy reported per study, 50–100%).
	**10b**	List and define all other variables for which data were sought (e.g. participant and intervention characteristics, funding). Describe assumptions about missing information.	**OK**<br>2.4
**Study risk of bias assessment**	**11**	Specify the methods used to assess risk of bias, including tool(s) used, how many reviewers assessed each study and whether independent, and automation tools.	**OK**<br>2.5, Table 1, Table 2 and 2.4 (inter-rater agreement Sheet 7: κ 0.83–1.00)

PRISMA

Section and Topic	Item #	Checklist item	Location where item is reported
**Effect measures**	**12**	Specify for each outcome the effect measure(s) used in the synthesis or presentation of results.	**OK**<br>2.6: accuracy classification as a representative measure; Mann–Whitney, Fisher, Spearman tests.
**Synthesis methods**	**13a**	Describe the processes used to decide which studies were eligible for each synthesis.	**OK**<br>2.6
	**13b**	Describe any methods required to prepare the data for presentation or synthesis (missing statistics, conversions).	**OK**<br>2.6: accuracy 50–100%.
	**13c**	Describe any methods used to tabulate or visually display results of individual studies and syntheses.	**OK**<br>2.6 y 3 (Tables 3–6; Figures 1–3).
	**13d**	Describe methods used to synthesize results and rationale. If meta-analysis, describe model(s), heterogeneity methods and software.	**OK**<br>No meta-analysis (narrative synthesis + inferential tests) was performed; described in 2.6 with RStudio.
	**13e**	Describe any methods used to explore possible causes of heterogeneity (subgroup analysis, meta-regression).	**OK**<br>2.6 and 3.3.1 (comparisons by technology and by model family; external validation by year).
	**13f**	Describe any sensitivity analyses conducted to assess robustness of the synthesized results.	**OK**<br>4.6 and Table 6.
**Reporting bias assessment**	**14**	Describe any methods used to assess risk of bias due to missing results in a synthesis (reporting biases).	**OK**<br>2.6 y 4.7, 3.4/Table 3, 4.7.
**Certainty assessment**	**15**	Describe any methods used to assess certainty (confidence) in the body of evidence for an outcome.	**OK**<br>ADDED to 2.6 (Methods): It is stated that GRADE was not applied because the outcomes are descriptive (frequency of external validation, deployment counts), and that certainty is conveyed through the risk of bias framework (2.5).
**RESULTS**			
**Study selection**	**16a**	Describe the results of the search and selection process, from records identified to studies included, ideally using a flow diagram.	**OK**<br>3.1 y Figure 1
	**16b**	Cite studies that might appear to meet the inclusion criteria, but which were excluded, and explain why.	**OK**<br>ADDED (3.1 / Figure 1).
**Study characteristics**	**17**	Cite each included study and present its characteristics.	**OK**<br>3.1 (general characteristics); characteristics by study in the 7-sheet workbook (supplementary).
**Risk of bias in studies**	**18**	Present assessments of risk of bias for each included study.	**OK**<br>3.7 (distribution of scores and risk of bias) + Sheet 7 of the supplementary workbook.
**Results of individual studies**	**19**	For all outcomes, present for each study summary statistics and an effect estimate with its precision (e.g. CI), ideally using tables or plots.	**OK**<br>3.4 and Table 4
**Results of syntheses**	**20a**	For each synthesis, briefly summarise the characteristics and risk of bias among contributing studies.	**OK**<br>3.3.1 and 3.4.1
	**20b**	Present results of all statistical syntheses. If meta-analysis, present summary estimate, precision and heterogeneity. If comparing groups, describe direction of effect.	**OK**<br>3.3.1 and Table 4

Section and Topic	Item #	Checklist item	Location where item is reported
	**20c**	Present results of all investigations of possible causes of heterogeneity among study results.	**OK**<br>3.3.1, Table 4 (Panel A, Panel C).
	**20d**	Present results of all sensitivity analyses conducted.	**OK**<br>4.6 and Table 6.
**Reporting biases**	**21**	Present assessments of risk of bias due to missing results (reporting biases) for each synthesis assessed.	**OK**<br>3.4 and Table 3
**Certainty of evidence**	**22**	Present assessments of certainty (confidence) in the body of evidence for each outcome assessed.	**OK**<br>3.4.1, 4.1 and §5
**DISCUSSION**			
**Discussion**	**23a**	Provide a general interpretation of the results in the context of other evidence.	**OK**<br>4.1 (The Detection–Prevention Gap) and 4.4.
	**23b**	Discuss any limitations of the evidence included in the review.	**OK**<br>4.2 and 4.3
	**23c**	Discuss any limitations of the review processes used.	**OK**<br>4.7 (Limitations of This Review).
	**23d**	Discuss implications of the results for practice, policy, and future research.	**OK**<br>4.5 (What Would Genuine Translation Look Like?) and 5 (Conclusions).
**OTHER INFORMATION**			
**Registration and protocol**	**24a**	Provide registration information, including register name and registration number, or state that the review was not registered.	**OK**<br>2.1: protocol deposited prospectively in Zenodo (DOI:10.5281/zenodo.20785718).
	**24b**	Indicate where the review protocol can be accessed, or state that a protocol was not prepared.	**OK**<br>2.1: protocol accessible via DOI Zenodo (10.5281/zenodo.20785718).
	**24c**	Describe and explain any amendments to information provided at registration or in the protocol.	**OK**<br>ADDED 2.1: "No amendments were made to the information provided in the registered protocol (Zenodo DOI: 10.5281/zenodo.20785718)."
**Support**	**25**	Describe sources of financial or non-financial support for the review, and the role of the funders or sponsors.	**OK**<br>ADDED (Funding): "This systematic review received no specific funding from public, commercial, or not-for-profit funding agencies."
**Competing interests**	**26**	Declare any competing interests of review authors.	**OK**<br>ADDED (Competing interests): "The authors declare no competing interests."
**Availability of data, code and other materials**	**27**	Report which of the following are publicly available and where: data collection forms; data extracted; data used for analyses; analytic code; other materials.	**OK**<br>2.1 (protocol in Zenodo) and references to Supplementary Table S7.

From: Page MJ, McKenzie JE, Bossuyt PM, Boutron I, Hoffmann TC, Mulrow CD, et al. The PRISMA 2020 statement: an updated guideline for reporting systematic reviews. BMJ 2021;372:n71. doi: 10.1136/bmj.n71. This work is licensed under CC BY 4.0. To view a copy of this license, visit https://creativecommons.org/licenses/by/4.0/.
